# Systems Toxicology

**DOI:** 10.17179/excli2015-762

**Published:** 2015-12-22

**Authors:** Ahmed Ghallab

**Affiliations:** 1 Forensic Medicine and Toxicology Department, Faculty of Veterinary Medicine, South Valley University, Qena, Egypt

## ⁯⁯⁯⁯⁯⁯

In recent years system-level understanding has become a cutting edge topic in toxicology (Geenen et al., 2012[11]; Marchan et al., 2012[22]; Widom et al., 2014[31]; Kell, 2010[20]). Recently, a definition of Systems Toxicology has been suggested: *“Systems Toxicology is the integration of classical toxicology with quantitative analysis of large networks of molecular and functional charges occurring across multiple levels of biological organization”* (Sturla et al., 2014[27]). Although this definition has been published by outstanding scientists in this field of research it leaves some questions open. Is “analysis of large networks” really an essential requirement of Systems Toxicology? It is out of question that understanding the interactions of different levels of biological organization is of high interest. However, is the analysis of “charges occurring across multiple levels of biological organization” another indispensible necessity of Systems Toxicology? And how is “classical toxicology” integrated “with quantitative analysis of large networks”? Does not already “classical toxicology” use quantitative methods and e. g. network analysis? I will stop here torturing the reader with further questions. The point I wish to make is that I feel sometimes a bit defeated by the awesome but not fully clear sentences in this field of research. Important, in my opinion, is whether Systems Toxicology leads to answers of questions that are otherwise difficult to obtain. This will be illustrated by three examples:

- The liver is known for its outstanding capacity to regenerate after toxic damage. Within a relatively short period of time millions of cells find their new position to restore functional tissue architecture. Until recently, little was known which mechanisms orchestrate this process (Drasdo et al., 2014[8][7]). In principle cytokines released from dead cells may be responsible. However, numerous further possibilities, e.g. oxygen gradients, cytokine release from stellate cells or Kupffer cells, etc., may alternatively play a role. However, Systems Toxicology based simulations demonstrated that a so far unknown mechanism, named “hepatocyte sinusoid alignment” (HAS) is crucial (Hoehme et al., 2007[19], 2010[18]; Hammad et al., 2014[17]). During HAS hepatocytes align in the direction of the endothelial cells of the sinusoids. Therefore, the endothelial cells control the architecture of the liver's sheets of hepatocytes and also give the critical stimuli to proliferate. This Systems Toxicology driven prediction of the key role of endothelial cells was later confirmed by knockout experiments (Ding et al., 2010[6], 2014[5]). The practical relevance for toxicology: as soon as sinusoidal endothelial cells are destroyed by chemicals the risk of fibrosis strongly increases.
- Recently, a Systems Toxicology approach with a metabolic model of ammonia metabolism predicted that under specific conditions of hyperammonemia, glutamate dehydrogenase (GDH) may switch its direction from ammonia production to ammonia consumption (Schliess et al., 2014[25]; Ghallab et al., 2015[12]). This discovery could be used for normalizing increased levels of ammonia in blood of mice by infusing GDH and its cofactors at optimized concentrations.
- Cholestasis in liver disease triggers proliferative responses of the biliary tree. With the help of systems simulations it could be shown that adaptive remodeling of interlobular bile ducts aims at optimizing the intraluminal surface area by corrugation and branching (Vartak et al., 2015[28]). This is part of a process to adapt to a situation where increased amounts of bile salts must be reabsorbed. Therefore, therapy of cholestasis should not aim at antagonizing proliferative responses of the biliary tree, because it represents an adaptive response to avoid organ failure.

Understanding the mechanisms of organ toxicity has always been a major goal in toxicology (Campos et al., 2014[3]; Hammad, 2014[17]; Godoy et al., 2015[16], 2013[15], 2012[13], 2009[14]; Dias da Silva et al., 2013[4]; Driessen et al., 2013[9]; Shimada et al., 2012[26]; Baulies et al., 2015[2]; Reif, 2014[23][24]). In many circumstances Systems Toxicology techniques may help to gain a deeper understanding, particularly in situations where several mechanisms interact and due to high complexity the situation is difficult to understand intuitively (Widera et al., 2014[30]; Friebel et al., 2015[10]; Bartl et al., 2015[1]). Nevertheless, it is important that clear hypotheses are addressed by the simulations and that model predictions can be confirmed or rejected experimentally.
